# Transcriptome Analysis of the Nematode *Caenorhabditis elegans* in Acidic Stress Environments

**DOI:** 10.3389/fphys.2020.01107

**Published:** 2020-09-10

**Authors:** Yanyi Cong, Hanwen Yang, Pengchi Zhang, Yusu Xie, Xuwen Cao, Liusuo Zhang

**Affiliations:** ^1^CAS Key Laboratory of Experimental Marine Biology, Institute of Oceanology, Chinese Academy of Sciences, Qingdao, China; ^2^Laboratory for Marine Biology and Biotechnology, Qingdao National Laboratory for Marine Science and Technology, Qingdao, China; ^3^Center for Ocean Mega-Science, Chinese Academy of Sciences, Qingdao, China; ^4^University of Chinese Academy of Sciences, Beijing, China

**Keywords:** pH stress, acidic environment, cuticle collagens, xenobiotic detoxification, P450, *Caenorhabditis elegans*

## Abstract

Ocean acidification and acid rain, caused by modern industries’ fossil fuel burning, lead to a decrease in the living environmental pH, which results in a series of negative effects on many organisms. However, the underlying mechanisms of animals’ response to acidic pH stress are largely unknown. In this study, we used the nematode *Caenorhabditis elegans* as an animal model to explore the regulatory mechanisms of organisms’ response to pH decline. Two major stress-responsive pathways were found through transcriptome analysis in acidic stress environments. First, when the pH dropped from 6.33 to 4.33, the worms responded to the pH stress by upregulation of the *col*, *nas*, and *dpy* genes, which are required for cuticle synthesis and structure integrity. Second, when the pH continued to decrease from 4.33, the metabolism of xenobiotics by cytochrome P450 pathway genes (*cyp*, *gst*, *ugt*, and ABC transporters) played a major role in protecting the nematodes from the toxic substances probably produced by the more acidic environment. At the same time, the slowing down of cuticle synthesis might be due to its insufficient protective ability. Moreover, the systematic regulation pattern we found in nematodes might also be applied to other invertebrate and vertebrate animals to survive in the changing pH environments. Thus, our data might lay the foundation to identify the master gene(s) responding and adapting to acidic pH stress in further studies, and might also provide new solutions to improve assessment and monitoring of ecological restoration outcomes, or generate novel genotypes via genome editing for restoring in challenging environments especially in the context of acidic stress through global climate change.

## Introduction

As an essential environmental factor, pH affects many life processes such as growth, development, metabolism, as well as immune regulation. Whether on land or in water, living organisms often experience pH fluctuations. In recent years, increasing carbon dioxide emissions contribute to ocean acidification and increase the challenges of living environment pressures faced by marine organisms. Due to the increase in H^+^, the marine chemical balance will be broken down, making a variety of marine organisms and even ecosystems that depend on the stability of the chemical environment face great threats (Cornwall and Eddy, 2015; Doubleday et al., 2018). For example, a decrease in the pH of seawater can seriously affect the survival and development of larvae of Atlantic cod, causing damage to important organs such as gills and liver (Stiasny et al., 2018). The decrease in pH has a negative impact on the metamorphosis of Pacific oyster larvae by downregulating several proteins involved in energy production, metabolism, and protein synthesis (Dineshram et al., 2016).

At the same time, an increase in the acidity of rain will also cause a drop in the pH of the soil. China has become the third largest zone of acid rain pollution in the world after North America and Europe (Rodhe et al., 2002). In 2018, 18.9% of cities received precipitations with an annual average pH lower than 5.6 (Ministry of Ecology and Environment of the People’s Republic of China, 2019), which means that around one in five cities in China is enveloped by acid rain. Soil acidification will change the composition and function of soil biological communities, reduce the diversity of soil animal communities (Liu et al., 2014; Wang et al., 2014; Wei et al., 2017), and inhibit the activities of soil animals (Zhang J. E. et al., 2015). Under low soil pH, seedling roots in wheat upregulate genes involved in developmental processes, immune system processes, multi-organism processes, positive regulation of biological processes, and metabolic processes of the biological processes. Meanwhile, it downregulates genes belonging to the molecular function category including transporter activity, antioxidant activity, and molecular transducer activity, and to the extracellular region of the cellular component category (Hu et al., 2018).

In addition, pH is one of the most frequent ecological factors in aquaculture. Many factors can lead to a decrease in the pH of the aquaculture water environment, such as the respiration of plants and plankton in the water, decomposition of organic matter, and discharge of acid industrial pollutants. Especially in the intensive aquaculture system, the change in pH is inevitable due to the increase in breeding density and the limitation of the water circulation. There is more organic matter accumulated in the deep sludge; hence, the pH value of the bottom water is generally the lowest (Delgado et al., 2003). When the pH in the water environment deviates from the appropriate range of aquatic animals, it will cause damage to the tissues and organs of the cultured organisms, resulting in the impact on its energy metabolism, immunity, growth, and development (Chen and Chen, 2003; Garcia Parra and Baldisserotto, 2007; Yu et al., 2015), causing serious economic losses to aquaculture industries (Jiang et al., 2016).

As mentioned above, the phenomenon of pH decline in many environmental changes is pervasive and widespread and will have a series of negative effects on living organisms. Therefore, studying the biology of acidic environment stress is significant. However, the molecular mechanisms of animals’ response to acidic pH stress are largely unknown.

*Caenorhabditis elegans* is widely used as a model organism for many reasons. It is the first multicellular organism to have its genome completely sequenced (C. elegans Sequencing Consortium, 1998), which facilitates bioinformatics analysis. Besides, its genetic tractability, ease of handling, and maintenance also make *C. elegans* a powerful model system to study development, neurobiology, and stress responses (Brenner, 1974; Corsi et al., 2015; Zhang L. et al., 2015; Hunt, 2016). Only two studies were performed to study pH response in *C. elegans*. One reported that *C. elegans* showed a wide range of pH tolerance (pH 3.1∼pH 11.9) (Khanna et al., 1997). The other showed that the *cah-4b* transcript, encoding a putative carbonic anhydrase, was upregulated under alkaline pH (Hall et al., 2008). However, the underlying mechanisms of *C. elegans*’ response to acidic pH stress are mostly mysteries.

In this study, we employed RNA-seq (RNA sequencing) to ask how *C. elegans* responds to the acidic pH stresses. We found that there might be two major strategies *C. elegans* used to deal with pH stress: first, the expression level of cuticle structure and integrity-related genes was significantly increased when pH declined from 6.33 to 4.33; second, to deal with even lower pH stress, *C. elegans* substantially increased their xenobiotic metabolism by cytochrome P450 pathway genes. Thus, cuticle structure reorganization and xenobiotic metabolism reprogramming might be key pathways in response to pH stress in nematodes. Furthermore, the mechanisms found in this study might be applied to other invertebrate and vertebrate animals. Thus, our data may lay a foundation for further exploring the molecular mechanisms of animals’ response and adaptation to acidic pH stress environment in the wild.

## Materials and Methods

### Strains

We obtained the *C. elegans* wild-type Bristol N2 strain from the Caenorhabditis Genetics Center (CGC). N2 worms were cultured on nematode growth medium (NGM) agar plates seeded with *E. coli* OP50 and maintained at 20°C (Brenner, 1974).

### Worm Synchronization

In order to obtain synchronized nematodes, the gravid hermaphrodites were treated with basic hypochlorite solution at room temperature until each adult worm was digested (Silva, 2005). Eggs were collected and cultured overnight on unseeded nematode growth medium agar NGM plates at 20°C until incubation for about 12 h.

### pH Viability Assay

Thirty-five synchronized newly hatched L1 stage N2 nematodes were cultured in seeded (10 μl OP50 per plate) 3-cm-diameter NGM pH plates (0.3 g NaCl, 1.7 g Agar, 0.25 g Peptone, 97.5 ml H_2_O, 0.1 ml M MgSO_4_, 0.1 ml M CaCl_2_, 0.1 ml 5 mg/ml cholesterol in ethanol buffered at the appropriate pH by varying hydrochloric acid and sodium hydroxide accordingly, 2.5 ml M K_2_HPO_4_ buffer solution was added to maintain a stable pH). The growth and development of worms were observed, and the earliest time of the worms’ egg laying observed on each pH plate was recorded (pH 2.53, pH 2.73, pH 2.93, pH 3.13, pH 3.33, pH 4.33, pH 5.33, pH 6.33, pH 7.33, pH 8.33, pH 9.33, pH 10.33, and pH 11.33, respectively). Three replicates were performed for each pH conditions.

### Preparation of RNA-Seq Libraries and Data Analysis

To obtain synchronized *C. elegans* newly hatched L1 larvae, embryos were obtained with standard hypochlorite treatment and letting the L1 larvae hatch and undergo growth arrest on unseeded plates (Silva, 2005). The synchronized L1 larvae (around 60,000 worms) were transferred to a seeded (100-μl OP50 per plate, coating the entire plate evenly with a coating stick) 9-cm-diameter pH plate. Three replicates were performed for each pH conditions (pH 2.93, pH 3.13, pH 4.33, and pH 6.33). The larvae were collected after incubating for 3 h at 20°C in different pH environments. The worms were washed with M9 three times to remove the bulk of the residual bacteria. Then the samples were transferred to 1.5-ml tubes, and the excess supernatants were removed via centrifugation (2,000 × *g*, 5 min). The samples were frozen immediately in liquid nitrogen for 5 min and stored at −80°C. After the sample was rapidly ground with liquid nitrogen, RNA was extracted according to the Trizol method. RNA integrity was analyzed by 1% agarose gel electrophoresis and Bioanalyzer 2100 system. RNA purity was checked using the NanoPhotometer^®^ spectrophotometer. Accurate quantification of RNA concentration was measured by the Qubit 2.0 Fluorometer. Sequencing libraries were generated using NEBNext^®^ Ultra^TM^ RNA Library Prep Kit for Illumina^®^ (NEB, United States) following manufacturer’s recommendations. qRT-PCR was performed to accurately quantify the effective concentration of the library to ensure library quality. Then, the libraries were sequenced on an Illumina Hiseq platform, and 150-bp paired-end reads were generated. Reference genome and gene model annotation files were downloaded from WormBase (WS269). An index of the reference genome was constructed using Hisat2 v2.0.5, and paired-end clean reads were aligned with the reference genome using Hisat2 v2.0.5 (Kim et al., 2015, 2019; Pertea et al., 2016). FeatureCounts v1.5.0-p3 was used to count the read numbers mapped to each gene (Liao et al., 2013). Then, the FPKM of each gene was calculated based on the length of the gene and read count mapped to this gene. Differential expression analysis of two groups was performed using the R package DESeq2 (1.16.1) based on the negative binomial distribution (Love et al., 2014). The Benjamini and Hochberg’s approach was used to adjust the resulting *P*-values to control the false discovery rate. Corrected value of *P* < 0.05 and fold change > 2 were set as the threshold for significantly differential expression. GO enrichment analysis and KEGG pathway enrichment analysis of differentially expressed genes were achieved by clusterProfiler R package with an adjusted value of *P* < 0.05 (Yu et al., 2012).

### Quantitative Real-Time PCR Validation

Some of the key genes of our interest were selected for qPCR validation (*col-91*, *col-146*, *col-39*, *nas-38*, *nas-1*, *dpy-1*, *dpy-5*, *cyp13A5*, *cyp13A12*, *gst-31*, *gst-37*, *ugt-56*, *ugt-66*, *haf-1*, *mrp-2*, *nhr-11*, *nhr-237*, *hsp-16.1*, *hsp-70*). Total RNA was obtained by the same method applied in the sample preparation of RNA-seq libraries. Three RNA samples were collected for each pH conditions (pH 2.93, pH 3.13, pH 4.33, and pH 6.33, three biological replicates, respectively). The ReverTra Ace qPCR RT Master Mix with gDNA Remover Kit (TOYOBO) was used for reverse transcription. The synthesized cDNA was diluted 1:40. Four microliters of the diluted cDNA was used to carry out qPCR with SYBR^®^ Green Realtime PCR Master mix on a QuantStudio Real-Time PCR System following the manufacturer’s protocol. Primer sequences are provided in Supplementary File 1. Genes tested from each biological replicate had three technical repeats. Each gene of interest was normalized to an internal control gene (*tba-1*) and expressed as a fold change of treatment groups (pH 4.33, pH 3.13, and pH 2.93) compared with the control group (pH 6.33), respectively. Statistical analysis was performed by applying a two-tailed *t*-test to compare the treatment groups and control group on GraphPad Prism 5.

## Results

### Egg Laying Time of *C. elegans* in Different pH Environments

**FIGURE 1:**
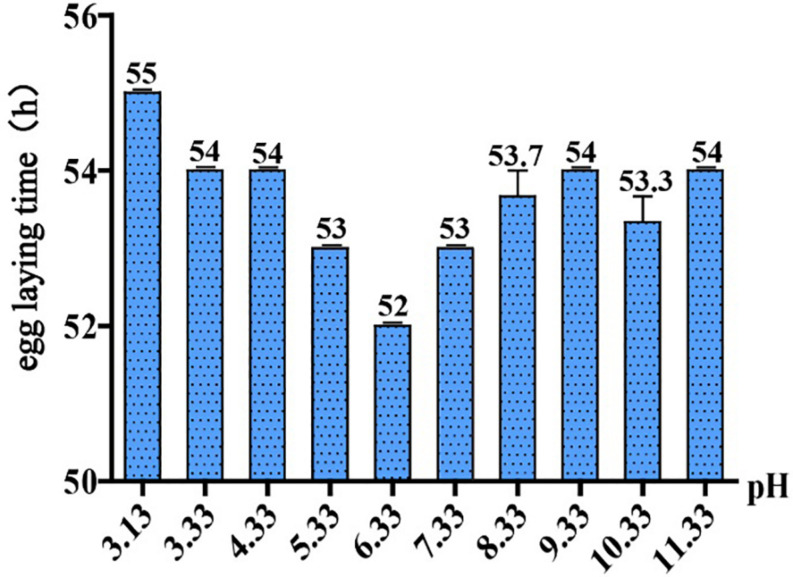
Egg laying time in different pH environments. Thirty-five newly hatched L1 were cultured at specific pH medium. The earliest observed egg laying time on each plate was recorded. Error bars represent the standard error from three replicated experiments.

To ask if pH stress affects *C. elegans* development and growth, we performed phenotypic experiments in different pH environments. The egg laying time of *C. elegans* is an easy-to-observe indicator, so it is feasible to judge the growth rate of *C. elegans* by its egg laying time. Our results showed that *C. elegans* is highly resistant to pH stress, and it can grow and reproduce in a wide range of pH (3.13–11.33) (Figure 1). The pH of the standard NGM was 6.33, and egg laying time was 52 h under this condition. Egg laying time was delayed for 54 h at pH 4.33 and pH 3.33, which was 2 h slower than the control group (pH 6.33). Egg laying time was 55 h at pH 3.13, which was 3 h slower than the control group (Figure 1). Strikingly, pH 2.93 and pH 3.13 only differed by 0.2, but this difference had a huge effect on the growth of N2. We noticed that *C. elegans* cannot lay eggs at pH 2.93; however, the nematode can grow and reproduce on the plate of pH 3.13, although with a 3-h delay in egg laying (Figure 1). The nematode was not able to survive on the pH 2.53 plate, while on the plate of pH 2.73, there were few surviving worms on the fifth day. Furthermore, we found that *C. elegans* had a wide range of adaptation to alkalinity, and the egg laying time is about 54 h at the range of pH 7.33–11.33 (Figure 1).

### Identification of Differentially Expressed Genes in *C. elegans* Exposed to Acidic Environments

**FIGURE 2:**
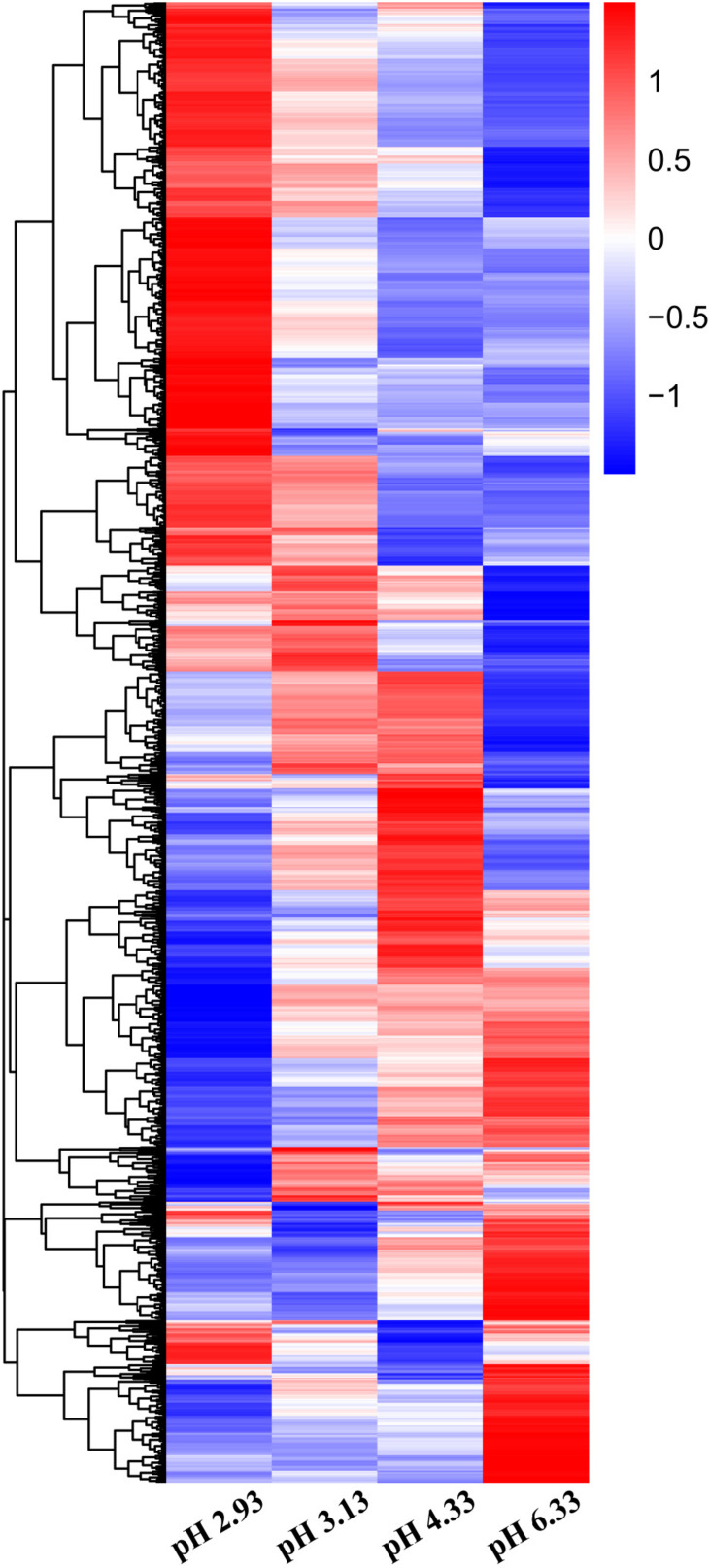
Heatmap of differentially expressed genes (DEGs) in *Caenorhabditis elegans* exposed to different acidic pH environments. The scale bar shows the *z*-score for a differentially expressed gene. Red indicates upregulation; blue indicates downregulation.

In this study, we used high-throughput RNA-seq to identify and quantify the differentially expressed genes (Figure 2). According to the data, we used pH 6.33 as the control group, pH 2.93, pH 3.13, pH 4.33 as the treatment group, FDR < 5% and fold change > 2 as the differential gene screening threshold. The number of upregulated genes and downregulated genes in each comparison combination is shown in Table 1. Details of significantly upregulated and downregulated differentially expressed genes in each condition are listed in Supplementary Files 2, 3.

**TABLE 1:** The number of differentially expressed genes.

pH comparisons	Total DEGs	DEGs up	DEGs down
4.33 vs. 6.33	449	317	132
3.13 vs. 6.33	648	404	244
2.93 vs. 6.33	1,131	662	469

*DEGs represent “differentially expressed genes.” They were identified using DESeq2 package (1.16.1) (FDR < 0.05 and FC > 2).*

### The Expression of Epidermal Synthesis-Related Genes Increased First and Then Decreased With the Decline in pH

**FIGURE 3:**
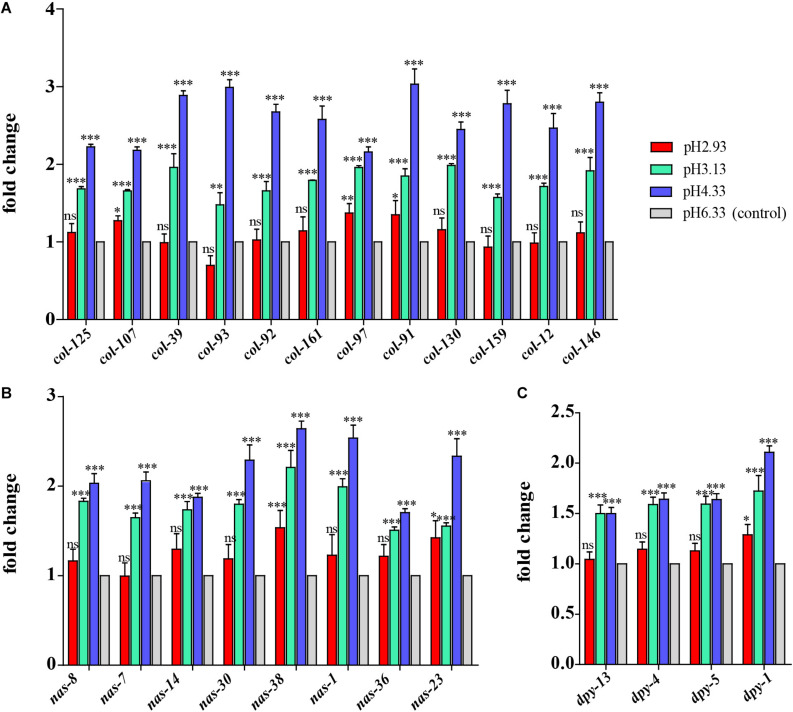
Expression of cuticle synthesis-related genes. **(A)** Transcript level of *col* genes. **(B)** Transcript level of *nas* genes. **(C)** Transcript level of *dpy* genes. Fold changes indicate the ratio of the treatment group (pH 2.93, pH 3.13, pH 4.33) to the control group (pH 6.33). The error bars represent standard error of the mean of three biological replicates per condition. ^∗^*P* < 0.05, ^∗∗^*P* < 0.01, ^∗∗∗^*P* < 0.001.

Based on GO analysis, we observed that the expression of genes related to epidermal synthesis varied gradually with the decrease in pH (Supplementary Files 4, 5). *col* and *dpy* encode the collagen genes and are supposed to be structural constituents of the cuticle. *nas* encodes an astacin-like metalloprotease and is predicted to have metalloendopeptidase activity (Stepek et al., 2011). It has been reported that *nas-35* is a gene closely related to epidermal synthesis (Novelli et al., 2004). *nas-1, -7, -14, -23*, and *-35* are all expressed in the hypodermis and probably process cuticle components like cuticular collagens (Park et al., 2010). The astacin metalloprotease *nas-36* and *nas-37* play an important role in cuticle ecdysis, which results in molting defects when mutated (Stepek et al., 2011). When the pH dropped from 6.33 to 4.33, *col*, *nas*, and *dpy* expressions were upregulated (Figure 3). When the pH continued to decrease from 4.33 to 3.13, the amplitude of the increase in the expression of these three genes decreased. When the pH dropped to 2.93, there was no significant difference compared to the expression levels detected at pH 6.33 (Figure 3). Thus, we propose that cuticle structure and integrity genes play the primary regulatory role to deal with acidic pH stress environments.

### Upregulation Genes Involved in the Metabolism of Xenobiotics by Cytochrome P450 Pathway

**FIGURE 4:**
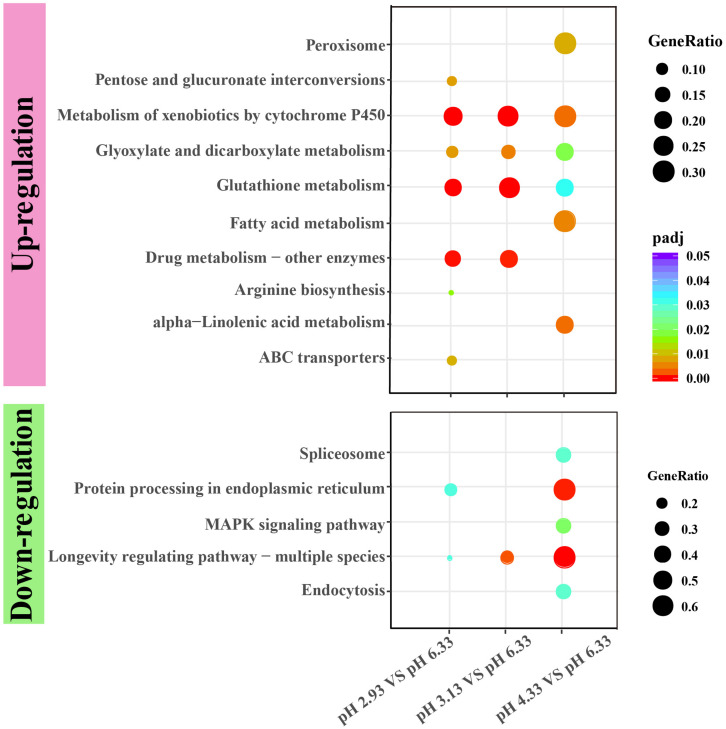
KEGG enrichment of upregulated and downregulated genes in each comparison. KEGG pathway enrichment analysis of differentially expressed genes was achieved by clusterProfiler R package with adjusted value of *P* < 0.05. The color from red to purple represents the significant size of the enrichment. Gene ratio is the ratio of the number of differential genes annotated on the KEGG pathway to the total number of differential genes.

**FIGURE 5:**
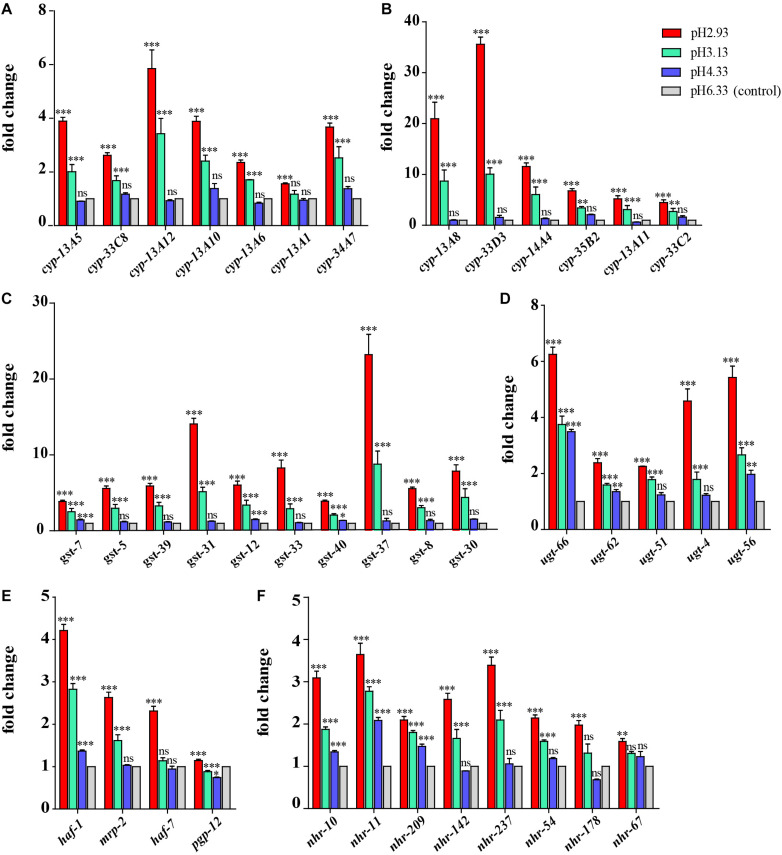
Expression of metabolism of xenobiotics by cytochrome P450 pathway-related genes and nuclear hormone receptor genes. **(A)** Transcript level of *cyp* genes. **(B)** Transcript level of *cyp* genes whose expression level is very low (hardly expressed) at pH 6.33 and pH 4.33. **(C)** Transcript level of *gst* genes. **(D)** Transcript level of *ugt* genes. **(E)** Transcript level of ABC transporter-related genes. **(F)** Transcript level of nuclear hormone receptor genes. Fold changes indicate the ratio of the treatment group (pH 2.93, pH 3.13, pH 4.33) to the control group (pH 6.33). The error bars represent standard error of the mean of three biological replicates per condition. ^∗^*P* < 0.05, ^∗∗^*P* < 0.01, ^∗∗∗^*P* < 0.001.

Through GO functional enrichment analysis and KEGG pathway analysis, it was found that when the pH decreases from 4.33 to 3.13, the metabolism of xenobiotics by cytochrome P450 pathway was significantly upregulated (Figure 4 and Supplementary Files 4, 5). Cytochrome P450 oxidase is the most important enzyme involved in the first phase of metabolism of xenobiotics, which is an iron porphyrin (erythrocyte heme) protein that catalyzes many types of redox reactions to increase the solubility of a substrate, usually by adding or uncovering a hydrophilic group (such as hydroxyl groups) (Supplementary Figure 1; Barnes, 1960). It can be seen that there was no significant change in the expression level of *cyp* gene when the pH dropped from 6.33 to 4.33 (Figure 5A) however, when the pH dropped to 3.13, the expression of the *cyp* gene was significantly upregulated (Figure 5A). Especially, we found six *cyp* genes (*cyp-13A8*, *cyp-33D3*, *cyp-14A4*, *cyp-35B2*, *cyp-13A11*, and *cyp-33C2*), whose expression levels were very low at pH 6.33 and pH 4.33, while these genes were significantly upregulated when the pH dropped to 3.13 (Figure 5B).

Similarly, when the pH dropped from 6.33 to 4.33, the expression levels of most *gst* and *ugt* genes were not changed significantly (except *gst-7*, *ugt-56*, *ugt-62*, *ugt-66*), However, when the pH dropped to 3.13, the expression levels of 10 *gst* and 5 *ugt* genes were significantly upregulated (Figures 5C,D). UDP-glucuronosyltransferase (UGT) and glutathione-S-transferase (GST) are two types of conjugating enzymes during phase II of the metabolism of xenobiotics. These two enzymes can catalyze the binding of xenobiotics with polar groups produced in phase I to glucuronic acid and glutathione, respectively, improving the water solubility of the phase I products, which facilitates them to discharge from the body (Supplementary Figure 1; Croom, 2012).

Besides, the gene expression levels of *haf-1* and *pgp-12* showed an increasing trend with the decreasing pH. When the pH decreased from 6.33 to 4.33, the expression levels of the *mrp-2* and *haf-7* genes were not changed significantly. However, when the pH dropped to 3.13, the expression level of the *mrp-2* gene was significantly upregulated. When the pH dropped to 2.93, the expression level of *haf-7* was significantly upregulated (Figure 5E). *haf-1*, *haf-7*, *mrp-2*, and *pgp-12* genes are ABC (ATP-binding cassette) transporters in *C. elegans*. The metabolites that have undergone the first and second phases of reactions are ultimately transported out of the cell by the ABC transporters.

In summary, we found that most xenobiotic detoxification pathway genes increased significantly in severe acidic pH stress environment, including P450 *cyp* genes in phase I, *ugt* and *gst* genes in phase II, and ABC transporters.

### Upregulation of Gene Expression of Nuclear Hormone Receptors

In addition to the P450 pathway, we found that the gene expression of nuclear hormone receptors also showed a similar trend. It can be seen that the gene expression of *nhr-10*, *nhr-11*, and *nhr-209* showed an upward trend when the pH declined (Figure 5F). When the pH dropped from 6.33 to 4.33, the expression levels of *nhr-142*, *nhr-237*, *nhr-54*, and *nhr-178* genes were not changed significantly. However, when the pH dropped to 3.13, the expression levels of these *nhr* genes were significantly upregulated (except *nhr-178*).

### Downregulation of Heat Shock Protein Gene Expression

**FIGURE 6:**
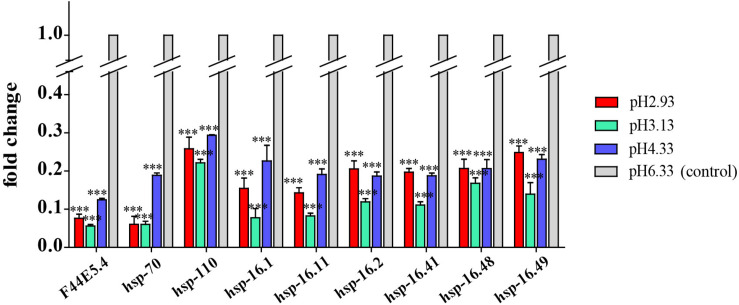
Transcript level of *hsp* genes. Fold changes indicate the ratio of the treatment group (pH 2.93, pH 3.13, pH 4.33) to the control group (pH 6.33). The error bars represent standard error of the mean of three biological replicates per condition. ^∗^*P* < 0.05, ^∗∗^*P* < 0.01, ^∗∗∗^*P* < 0.001.

As a common indicator of cellular stress, heat shock proteins are induced to express under many stimuli such as cold, infiltration, drought, salt, ultraviolet light, oxidative stress, and pathogen infection (Swindell et al., 2007). Unexpectedly, our results showed that the expression of heat shock protein genes was significantly downregulated in acidic pH environment (Figure 6). In this study, these downregulated heat shock protein genes were enriched by protein processing in the endoplasmic reticulum and the longevity regulated pathway by the KEGG pathway analysis (Figure 4). Our results suggested that acidic pH stress might severely affect the capacity of protein processing in *C. elegans*.

### Quantitative Real-Time PCR Validation

**FIGURE 7:**
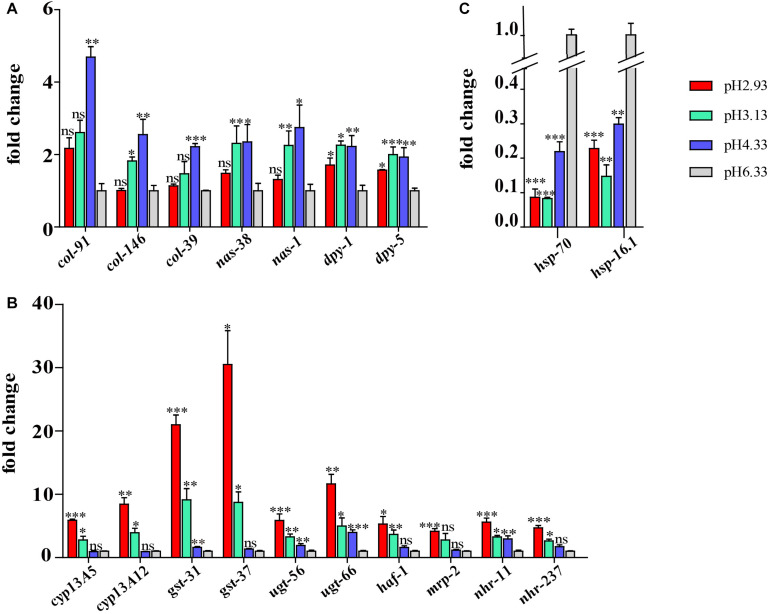
Validation of the RNA sequencing (RNA-seq) results using quantitative real-time (qRT)-PCR. **(A)** Expression level of cuticle structure and integrity-related genes. **(B)** Expression level of xenobiotic metabolism by cytochrome P450 pathway genes. **(C)** Expression level of *hsp* genes. Fold change indicates the ratio of the treatment group (pH 2.93, pH 3.13, pH 4.33) to the control group (pH 6.33). The mean fold changes and standard error of the mean of three biological replicates are graphed. ^∗^*P* < 0.05, ^∗∗^*P* < 0.01, ^∗∗∗^*P* < 0.001.

Some of the genes of interest were validated by qPCR. A similar trend was displayed in both the qRT-PCR and RNA-seq analyses. As shown in Figure 7A, the expression level of *col*, *nas*, and *dpy* genes increased significantly at pH 4.33 and then decreased with the decline in pH. Consistent with our RNA-seq data, the expression of the *cyp*, *gst*, *ugt*, *haf*, and *nhr* genes was increased significantly at pH 3.13 and 2.93 (Figure 7B). In addition, we confirmed that *hsp-70* and *hsp-16.1* genes were downregulated in all the acidic environments (Figure 7C).

## Discussion

As an important ecological factor, pH has a direct impact on the life processes of living organisms. Studying the mechanism of pH stress sensation and responding signaling in *C. elegans* might lay a foundation in understanding how invertebrate animals respond and adapt to environmental pH stresses. The decrease in environmental pH will force *C. elegans* to adjust the acid–base balance in the body, but the adjustment ability is limited. When facing a severe acidic environment, the growth and reproduction processes of *C. elegans* will be significantly affected; for instance, the egg laying time (the time required for *C. elegans* to develop from L1 larvae to adulthood laying the first few eggs) was delayed by 3 h at pH 3.13. Under the condition of pH 2.93, *C. elegans* could hardly lay eggs, or egg laying is much delayed. Acid–base adjustment also requires energy consumption. In order to adapt to the decrease in environmental pH, organisms transfer energy from growth and reproduction to ensure that basic metabolic processes are maintained (Pörtner et al., 2000; Pörtner, 2008), so their growth and development rate will slow down. We would like to make it clear that it is the impact of environmental pH on nematode development that is being investigated, while the impact on the internal pH of the nematode is unknown in this project.

### Cuticle Remodeling Genes Are Induced by Low pH

The nematode cuticle is a highly structured extracellular matrix composed predominantly of cross-linked collagens, additional insoluble proteins termed cuticlins, associated glycoproteins, and lipids (Page and Winter, 2003). It forms a hydrostatic skeleton and acts as a primary barrier and first line of defense against many environmental violations. Cuticle collagen synthesis pathway involves numerous co- and post-translational modification, processing, secretion, and cross-linking steps that, in turn, are catalyzed by specific enzymes and chaperones including astacin metalloprotease (Page and Winter, 2003). Nematode astacins are the key to the development of *C. elegans* and have special roles in hatching, molting, and cuticle synthesis (Hishida et al., 1996; Page and Winter, 2003; Stepek et al., 2011, 2015). Loss-of-function mutations in *dpy-31*/*nas-35* lead to typical cuticle synthesis defects (Novelli et al., 2004). The *nas-6*; *nas-7* double mutant grows slowly and has defects in the pharyngeal grinder, which is a cuticular structure important for food processing (Park et al., 2010).

*dpy-2*, *dpy-3*, *dpy-7*, and *dpy-10* are necessary for the formation of specific bands of collagen called annular furrows. When these genes are knocked down, annular furrows disappear accordingly, and the detoxification, hyperosmotic, and antimicrobial responses will be activated (Dodd et al., 2018). It is believed that annular furrows are damage sensors for detoxification, and hyperosmotic and antibacterial reactions (Dodd et al., 2018). It was reported that *dpy-1*, *dpy-5*, and *dpy-13* are required for alae (lateral ridges in the cuticle of the adult *C. elegans*) formation (Thein et al., 2003; Dodd et al., 2018). Based on our results, we speculate that alae is more sensitive when exposed to acidic pH stress because the expression of alae-related genes changes significantly at pH 4.33 (Figure 3C), while furrow-related genes are not significantly downregulated until pH 2.93 (Supplementary Figure 2).

Cuticle structure and integrity genes might play a primary regulatory role to deal with acidic pH stress environments. When the pH drops from 6.33 to 4.33, *C. elegans* is likely to fight lower pH stress by enhancing the synthesis and secretion of epidermal collagen, so the expression of *col*, *nas*, and *dpy* genes are upregulated. When the pH continues to decrease from 4.33, the amplitude of the increase in the expression of these genes decreases, which suggests that the damage caused by a more acidic environment is too strong to be handled via cuticle physical changes. It has been reported that cuticle structure and integrity, especially annular furrows, are damage sensors in certain abiotic and biotic stresses, such as osmotic, xenobiotic, and bacterial infection (Dodd et al., 2018).

### Metabolism of Xenobiotics by Cytochrome P450 Pathway Plays a Major Role in Extreme Acidic pH Stress Environments

Our results showed that as the pH decreases, genes involved in the metabolism of xenobiotics by cytochrome P450 pathway were all upregulated, and the overall trend was that the expression levels were not changed significantly when the pH dropped from 6.33 to 4.33. As the pH continued to drop, the expression of genes in this pathway was significantly upregulated (Figure 5). We propose that the pH decline causes different degrees of acidity pressure on the nematodes, which may lead to the accumulation of toxic substances in the nematode and triggers the upregulation of multiple genes in the cytochrome P450 pathway. Thus, the cytochrome P450 pathway might be a key metabolic pathway for biological response to severe acidity stress.

When the pH declined from 8.15 to 7.81, the cytochrome P450-like transcript of the great spider crab *Hyas araneus* was upregulated by 9.4-fold (Harms et al., 2014). Similarly, (Timmins-Schiffman et al., 2014) found in the proteomic analysis of the Pacific oysters (*Crassostrea gigas*) that when the pH fell from 8.0 to 7.3, the expression of cytochrome P450 1A5 was elevated by 2.7 times, and the expression of glutathione S-transferase Ω-1 was increased by 3.6 times, which is presumed to be caused by cellular antioxidant stress.

It was reported that exposure of *C. elegans* to hyperosmotic conditions can also lead to upregulation of the transcriptome of the detoxification pathway genes (glycosyltransferase, cytochrome P450, glutathione S-transferase, and ABC transporters), and their upregulations were partially dependent on SKN-1 (Dodd et al., 2018). *skn-1* (RNAi) reduces the expression of many phase II detoxification genes, especially within the *gst* gene class (Fukushige et al., 2017; Dodd et al., 2018). There are also substances that induce upregulation of the detoxification pathway genes independent of *skn-1*, for example, t-BOOH (tert-Butyl hydroperoxide), a stable lipid-soluble hydrogen peroxide that attacks lipids and proteins (Oliveira et al., 2009). In our study, the expression level of *skn-1* does not show significant difference among different pH conditions (Supplementary Figure 3). Thus, the upregulation of the detoxification pathway caused by acid stress is more likely to be independent of *skn-1* regulation in *C. elegans*.

Furthermore, we found that the expression of nuclear hormone receptor genes showed similar trends with xenobiotic detoxification genes in response to acidic pH stress (Figure 5F). Nuclear hormone receptors are important transcriptional regulators involved in a variety of physiological functions, many of which are associated with diseases such as cancer, diabetes, or hormone resistance syndromes. NHRs have broad substrate specificities in regulating detoxification enzymes, and they are critical hubs for the metabolism of endo- and xenobiotics (Hoffmann and Partridge, 2015). They also modulate the homeostasis of steroid hormones, other endogenous cholesterol derivatives, and lipid metabolism (Hoffmann and Partridge, 2015).

A recent study finds that *nhr-10* and *nhr-68*, both transcriptionally and functionally, are important in activating shunt gene expression in response to the excessive accumulation of propionate (Bulcha et al., 2019). In *C. elegans*, *nhr-8* has been demonstrated to regulate xenobiotic detoxification, which is necessary for anti-colchicine and chloroquine toxins (Lindblom et al., 2001). The activation of detoxification and immune response to mitochondrial toxin or pathogenic *Pseudomonas aeruginosa* is transcriptionally mediated by *nhr-45* (Mao et al., 2019). The NHR superfamily has conserved and typical structures, the DNA binding domain (DBD) and the ligand binding domain (LBD). Fifteen NHRs are highly conserved in *C. elegans*, Drosophila, and mammals. For example, the identity between the DBD of *C. elegans* NHR-67 and Drosophila DmTLL and human HsTLX was 79 and 70%, respectively (Sluder et al., 1999; Maglich et al., 2001). NHR-67 plays an essential role in larval development and functions as a component of a complex regulatory network that regulates the differentiation and organogenesis of vulva, thereby regulating egg laying (Fernandes and Sternberg, 2007). The identified NHR ligands are generally lipophilic including steroid hormones, glucocorticoids, and exogenous drugs (McEwan, 2009; Huang et al., 2010).

In this study, the expression pattern of *nhr* genes is highly consistent with that of the metabolism of xenobiotics by cytochrome P450 pathway genes, indicating that the xenobiotics are likely to bind to the nuclear hormone receptors directly or indirectly through the ligands, and then activate the upregulation of the detoxification-related genes of the xenobiotics.

Xenobiotic detoxification genes are reported in other animals’ responses to pH stress, although only one or few genes are found (Harms et al., 2014; Timmins-Schiffman et al., 2014). However, our data show a systematic pathway gene increase in acidic stress conditions. It has been reported that xenobiotic detoxification genes are involved in osmotic stress (Dodd et al., 2018), heat-shock stress (Joshi et al., 2016), and hypoxia stress environment (Leiser et al., 2015). According to our data, metabolism of xenobiotics by cytochrome P450 pathway might be a universal signaling pathway to deal with stress environments. Therefore, manipulating xenobiotic detoxification genes, through gene overexpression or CRISPR genome editing, might provide a solution to agriculture and ecosystem restoration in the global climate change contexts. Furthermore, our findings might be important for toxicology and pharmacological screenings, as drugs can significantly change the medium pH.

### Downregulation of Heat Shock Protein Genes

We observed that the expression of heat shock proteins was significantly downregulated in response to acidic environments (Figure 6). *F44E5.4*, *hsp-70*, and *hsp-110* all encode HSP70 heat shock protein family members. As a molecular chaperone, HSP70 promotes protein folding, membrane translocation, degradation of misfolded proteins, and protection of cells from stress-induced damage (Liu and Chen, 2013; Goncalves et al., 2017). HSP-110 is an ortholog of the human HSPA4. Rampelt et al. (2012) found that knock-down of *Caenorhabditis elegans hsp110* disrupted the dissolution of heat-induced protein aggregates and severely shortened the lifespan after heat shock.

*hsp-16.1*, *hsp-16.11*, *hsp-16.2*, *hsp-16.41*, *hsp-16.48*, and *hsp-16.49* all encode 16-kD heat shock protein that are members of the HSP16/HSP20 family of heat shock proteins. HSP-16.1 and HSP-16.41 are required for an acquired tolerance to heat stroke. HSP-16.1 is localized to the Golgi and functions with PMR-1/PMR1 Ca^2+^ and Mn^2+^ transporting ATPase and NUCB-1 to maintain calcium homeostasis under heat stroke (Kourtis et al., 2012). HSP-16.2 was a chaperone biomarker of lifespan in *C. elegans* (Burnaevskiy et al., 2019).

Therefore, stable expression of heat shock proteins might be necessary to maintain healthy physiological conditions. In our study, the expression of heat shock protein decreases with declined pH, which is consistent with the phenotype of the negative effects of pH drop on the growth and reproduction of the nematodes.

In conclusion, our results suggested that cuticle synthesis and integrity, together with the reprogrammed xenobiotic metabolism by cytochrome P450 pathway, were two major stress-responsive pathways for *C. elegans* in acidic stress environments. On one hand, when the pH drops from 6.33 to 4.33, the worms resist the damage caused by the acid culture plate by enhancing the synthesis and secretion of epidermal collagen. On the other hand, when the pH continues to decrease from 4.33, the metabolism of xenobiotics by the cytochrome P450 pathway genes plays a major role in protecting the nematodes from the toxic substances produced by the more acidic environment. At the same time, cuticle synthesis slows down possibly because of insufficient protective ability. To the best of our knowledge, this study is the first to systematically discover that there are two strategies in which *C. elegans* respond to acidic pH stress environment: cuticle structure reorganization and xenobiotic metabolism reprogramming. Furthermore, the mechanisms found in nematode might be also applied to other invertebrate and vertebrate animals to survive in the changing pH stress environments. Thus, our data might lay the foundation to identify the key gene(s) responding and adapting to acidic pH stress in further studies, and might also provide new solutions to improve assessment and monitoring of ecological restoration outcomes, or generate novel genotypes via genome editing for restoring in challenging environments especially in the context of acidic stress through global climate change.

## Data Availability Statement

The datasets generated for this study can be found in NCBI, and the BioProject ID: PRJNA649349.

## Author Contributions

YC and LZ conceived and designed the experiments. YC carried out the most of the experiments, analyzed the data, and wrote the manuscript. HY and PZ performed the RT-qPCR validation. YX and XC were involved in sampling and data analysis. LZ edited the manuscript and supervised the project. All authors read and approved the final manuscript.

## Conflict of Interest

The authors declare that the research was conducted in the absence of any commercial or financial relationships that could be construed as a potential conflict of interest.
