# Supplementary material for: Transcriptome Analysis of the Nematode Caenorhabditis elegans in Acidic Stress Environments
Source: Front Physiol. 2020 Sep 10;11:1107. doi: 10.3389/fphys.2020.01107 (PMC7511720; doi:10.3389/fphys.2020.01107)
Supplement: Supplementary file 2 [file Data_Sheet_2.PDF]

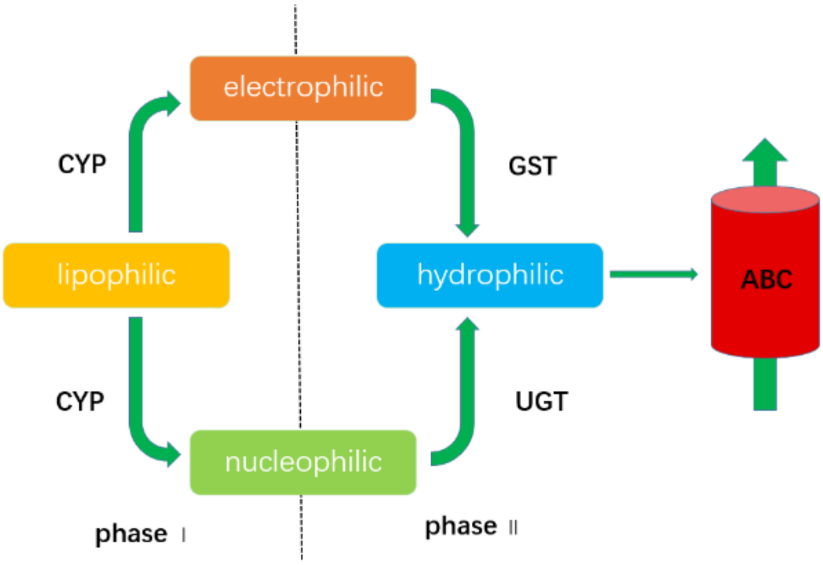


**Supplementary Figure 1.** Xenobiotics metabolism by cytochrome P450 pathway. In Phase 1, lipophilic xenobiotics are metabolized to electrophilic or nucleophilic substances by phase I enzymes such as Cytochrome P450s (CYPs). In phase II, the metabolites produced by the first-stage reaction are combined with polar ligands such as glucuronic acid, glutathione etc. under the catalysis of transferases (GST, UGT, etc.). Finally, these trait-changed compounds are transported extracellularly by the ABC transporter (Barnes, 1960; Croom, 2012; Dieterich et al., 2008)

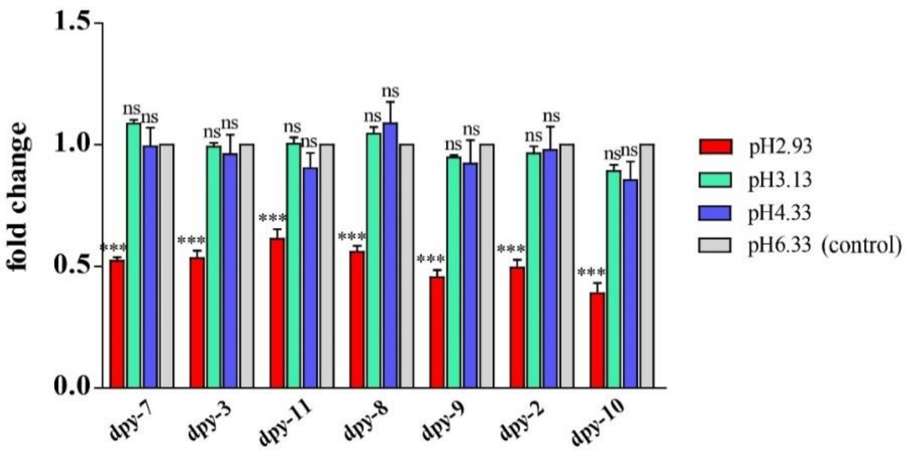


**Supplementary Figure 2.** Transcript level of *dpy* genes. Fold changes indicates the ratio of the treatment group (pH 2.93, pH 3.13, pH 4.33) to the control group (pH 6.33). The error bars represent standard error of the mean of three biological replicates per condition. **P* <0.05, ***P* <0.01, ****P* <0.001

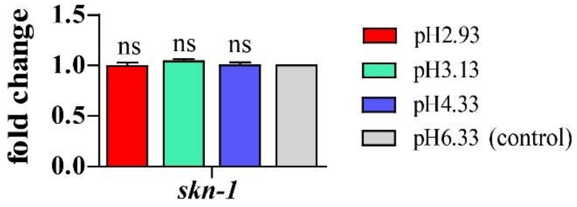


**Supplementary Figure 3.** Transcript level of *skn-1* gene. Fold changes indicates the ratio of the treatment group (pH 2.93, pH 3.13, pH 4.33) to the control group (pH 6.33). The error bars represent standard error of the mean of three biological replicates per condition. *$P$ <0.05, **$P$ <0.01, ***$P$ <0.001
